# Impact of age on frailty in liver cirrhosis: a prospective cohort study

**DOI:** 10.1007/s10238-025-01747-3

**Published:** 2025-06-13

**Authors:** Omkolsoum Alhaddad, Maha Elsabaawy, Asmaa Abedelhai, Gamal Badra, Marwa Elfayoumy

**Affiliations:** Department of Hepatology and Gastroenterology, National Liver Institute, Menoufia University, Shebeen Elkoom, Menoufia Egypt

**Keywords:** Frailty, Liver cirrhosis, Aging, Sarcopenia, Child–Pugh score

## Abstract

Frailty is an emerging predictor of adverse outcomes in liver cirrhosis, yet the interplay between aging and liver disease severity in driving frailty remains insufficiently understood. To evaluate the impact of age on the prevalence, severity, and predictors of frailty in patients with liver cirrhosis. In this prospective observational study, 460 adults with liver cirrhosis were assessed for frailty using the CFS (Clinical Frailty Scale) (CFS). Patients were classified as frail (CFS > 4) (210 cases), or non-frail (CFS ≤ 4) (250 cases). Demographic, clinical, and biochemical data of frail cases were collected. Multivariate and logistic regression analyses were performed to identify independent predictors of frailty. Frailty prevalence increased markedly with age—from 42% in patients aged 50–59 to over 90% in those aged ≥ 70. Age was moderately correlated with frailty (r = 0.40, *p* < 0.001). In multivariate analysis, both age (β = 0.0636, *p* < 0.001) and Child–Pugh score (β = 0.7874, *p* < 0.001) were independent predictors of frailty. Logistic regression (including interaction terms where appropriate) confirmed that each additional year of age increased frailty risk (OR = 1.13; 95% CI: 1.09–1.17, *p* < 0.001). Frailty in cirrhosis is strongly age-associated but also driven by hepatic dysfunction. These findings highlight the inadequacy of MELD-Na scores alone in capturing patient vulnerability, particularly in older adults. Future longitudinal studies and targeted prehabilitation strategies are warranted to mitigate frailty and improve outcomes in this vulnerable population.

## Introduction

Frailty is a clinical syndrome characterized by a decline in physiological reserves, leading to increased vulnerability to stressors and adverse health outcomes [1]. In patients with liver cirrhosis, frailty emerges as a critical determinant of morbidity, mortality, and liver transplant outcomes [2]. Unlike traditional models that focus solely on hepatic function, frailty assessment provides a holistic evaluation of a patient’s overall functional status, muscle strength, and resilience to disease progression [3].

Aging is a major factor influencing frailty, and older adults with cirrhosis are at a disproportionately higher risk of developing frailty due to age-related sarcopenia, comorbidities, and systemic inflammation [4]. However, frailty is not exclusive to elderly patients, as younger cirrhotic individuals may also exhibit features of frailty due to chronic liver disease progression, malnutrition, and metabolic dysfunction [5]. The interaction between age and frailty in cirrhotic patients remains incompletely understood, particularly in diverse patient populations with varying etiologies of liver disease.

Several studies have highlighted the impact of frailty on clinical outcomes in cirrhosis, including increased risk of hospitalization, infections, hepatic decompensation, and mortality [6, 7]. Traditional risk stratification tools such as the Model for End-Stage Liver Disease (MELD) and Child–Pugh classification focus primarily on liver function and fail to incorporate functional capacity and sarcopenia-related risk factors. Consequently, frailty assessment is gaining recognition as an independent predictor of adverse outcomes and transplant candidacy in cirrhotic patients [8].

Despite the growing body of evidence supporting frailty assessment in cirrhosis, age-specific differences in frailty prevalence, severity, and clinical impact have not been fully elucidated. It remains unclear whether frailty is predominantly an age-related phenomenon or if younger cirrhotic patients can develop frailty due to disease-driven muscle wasting and systemic dysfunction. Understanding this relationship is crucial for tailoring interventions, optimizing risk prediction models, and improving clinical management strategies for patients with cirrhosis.

This study aims to evaluate the impact of age on frailty in patients with liver cirrhosis, examining the prevalence, severity, and clinical implications across different age groups. By investigating the interaction between age and frailty, we seek to provide insights into early detection strategies, risk stratification models, and personalized management approaches for cirrhotic patients at risk of poor outcomes.

## Patients and methods

This prospective observational study was conducted at the National Liver Institute (NLI), Menoufia University, Egypt, between September 15, 2023, and June 15, 2024. The study aimed to evaluate the impact of age on frailty in adult patients with liver cirrhosis. Liver cirrhosis was diagnosed through a combination of clinical, laboratory, and radiological findings, and in selected cases, histopathological confirmation [9].

Eligible participants were adults aged 18 years or older with either compensated or decompensated cirrhosis. Patients were excluded if they had grade III–IV hepatic encephalopathy or significant cognitive impairment that could interfere with frailty assessment, active malignancy (except hepatocellular carcinoma within Milan criteria), acute liver failure or acute-on-chronic liver failure at presentation, recent hospitalization within the previous 30 days, or ICU admission. Patients with non-cirrhotic chronic liver diseases were also excluded.

Frailty was assessed at baseline using the Clinical Frailty Scale (CFS), a 9-point judgment-based tool that evaluates physical function, comorbidity burden, and level of independence. Trained clinicians, blinded to laboratory parameters, performed the CFS assessments to reduce observer bias. Patients were categorized into frail (CFS > 4) and non-frail (CFS ≤ 4) groups. The CFS is a validated tool in cirrhotic populations, with proven utility in predicting hospitalization, mortality, and transplant outcomes [10, 11].

Baseline demographic, clinical, and laboratory data were collected, including age, sex, body mass index (BMI), and comorbid conditions such as diabetes mellitus (diagnosed by HbA1c ≥ 6.5% or treatment history), hypertension, and cardiovascular disease. Liver disease severity was evaluated using the Child–Pugh classification (Classes A, B, or C) and the Model for End-Stage Liver Disease with sodium (MELD-Na) score.

*The sample size* was estimated based on an expected frailty prevalence of approximately 45.7%, with an 80% power and a 5% significance level. Accounting for possible attrition, the required sample size was calculated to be between 300 and 400 patients, leading to a final enrollment of 460 participants.

*Statistical analyses* were performed using IBM SPSS Statistics version 26.0 (2019). Continuous variables were presented as mean ± standard deviation (SD) or median with interquartile range (IQR), depending on normality of distribution, and compared using either the Student’s t-test or the Mann–Whitney U test. Categorical variables were compared using the Chi-square test or Fisher’s exact test. The relationship between age and frailty scores was evaluated using Pearson’s or Spearman’s correlation coefficients. To identify independent predictors of frailty, multivariate linear regression models were constructed, adjusting for age, Child–Pugh score, and diabetes status. Interaction terms, including age × diabetes, were tested for significance. Additionally, logistic regression analysis was used to evaluate the effect of age and other covariates on the likelihood of frailty (CFS > 4), and results were reported as odds ratios (OR) with 95% confidence intervals (CI). A p-value < 0.05 was considered statistically significant.

The study was approved by the Institutional Review Board (IRB) of the National Liver Institute under approval number 0014014FWA00034015. Written informed consent was obtained from all participants before their inclusion in the study, in compliance with the ethical standards of the Declaration of Helsinki.

## Results

In this prospective cohort study, we evaluated frailty in 460 patients with liver cirrhosis using the CFS (Clinical Frailty Scale) (CFS), classifying 210 (45.7%) as frail (CFS > 4) and 250 (54.3%) as non-frail (CFS ≤ 4). The frail cohort had a median age of 61 years (IQR: 55–66), with a balanced sex distribution (51% male) and a predominant etiology of hepatitis C virus (HCV) infection (56.2%) (Table 1).

**Table 1:** Baseline characteristics of frail patients with liver cirrhosis (n = 210)

Variable	Frail (CFS ≥ 5, n = 210)	Non-Frail (CFS < 5, n = 250)	*p*-value
Demographics			
Age (years, mean ± SD)	63.5 ± 8.7	60.4 ± 9.8	< 0.01
Sex, Male (%)	147 (70.0%)	187 (74.8%)	0.27
Clinical Characteristics			
Cause of Liver Disease			0.19
HCV (%)	161 (76.7%)	174 (69.6%)	
HBV (%)	12 (5.7%)	16 (6.4%)	
NBNC (%)	37 (17.6%)	60 (24.0%)	
Diabetes, yes (%)	68 (32.4%)	65 (26.0%)	0.14
Hypertension, yes (%)	54 (25.7%)	62 (24.8%)	0.83
BMI Category			0.02
Average (A, %)	86 (41.0%)	136 (54.4%)	
Overweight (O, %)	56 (26.7%)	61 (24.4%)	
Underweight (U, %)	39 (18.6%)	25 (10.0%)	
Obese (%)	29 (13.8%)	28 (11.2%)	
Liver Function			
MELD Score (mean ± SD)	10.2 ± 6.2	7.8 ± 5.1	< 0.001
Albumin (g/dL, mean ± SD)	3.5 ± 0.7	3.7 ± 0.7	< 0.01
Bilirubin (mg/dL, mean ± SD)	1.4 ± 1.4	1.2 ± 1.0	0.07
INR (mean ± SD)	1.3 ± 0.7	1.2 ± 0.3	0.03
ALBI Grade			< 0.001
Grade A (%)	153 (72.9%)	211 (84.4%)	
Grade B (%)	50 (23.8%)	36 (14.4%)	
Grade C (%)	7 (3.3%)	3 (1.2%)	
Child–Pugh Score (mean ± SD)	5.7 ± 1.3	4.3 ± 1.1	< 0.001
Complications			
Ascites/Lower Limb Edema, yes (%)	77 (36.7%)	46 (18.4%)	< 0.001

Frail defined as CFS ≥ 5 (n = 210), non-frail as CFS < 5 (n = 250). MELD-Na calculated for n = 459 due to one invalid entry. Ascites/edema includes MILD cases as Yes. *P*-values from Student’s t-tests (continuous), Chi-square test (categorical), or ANOVA (multi-category, e.g., ALBI, BMI). Significant differences (*p* < 0.05) in age, MELD, albumin, international normalized ratio (INR), Child–Pugh, ALBI grade, BMI, and ascites/edema. Sodium assumed at 135 mg/dL for MELD-Na consistency. BMI categories: A (18.5–24.9 kg/m^2^), O (25–29.9), U (< 18.5), OBESE (≥ 30)

CFS, CFS (Clinical Frailty Scale); DM, diabetes mellitus; HTN, hypertension; HCV, hepatitis C virus; HBV, hepatitis B virus; LL, lower limb; TB, total bilirubin; ALB, albumin; international normalized ratio (INR), international normalized ratio; Cr, creatinine; CP, Child–Pugh; MELD, Model for End-Stage Liver Disease; ALBI, Albumin-Bilirubin

### Frailty prevalence and its relationship with age

**Fig. 1:**
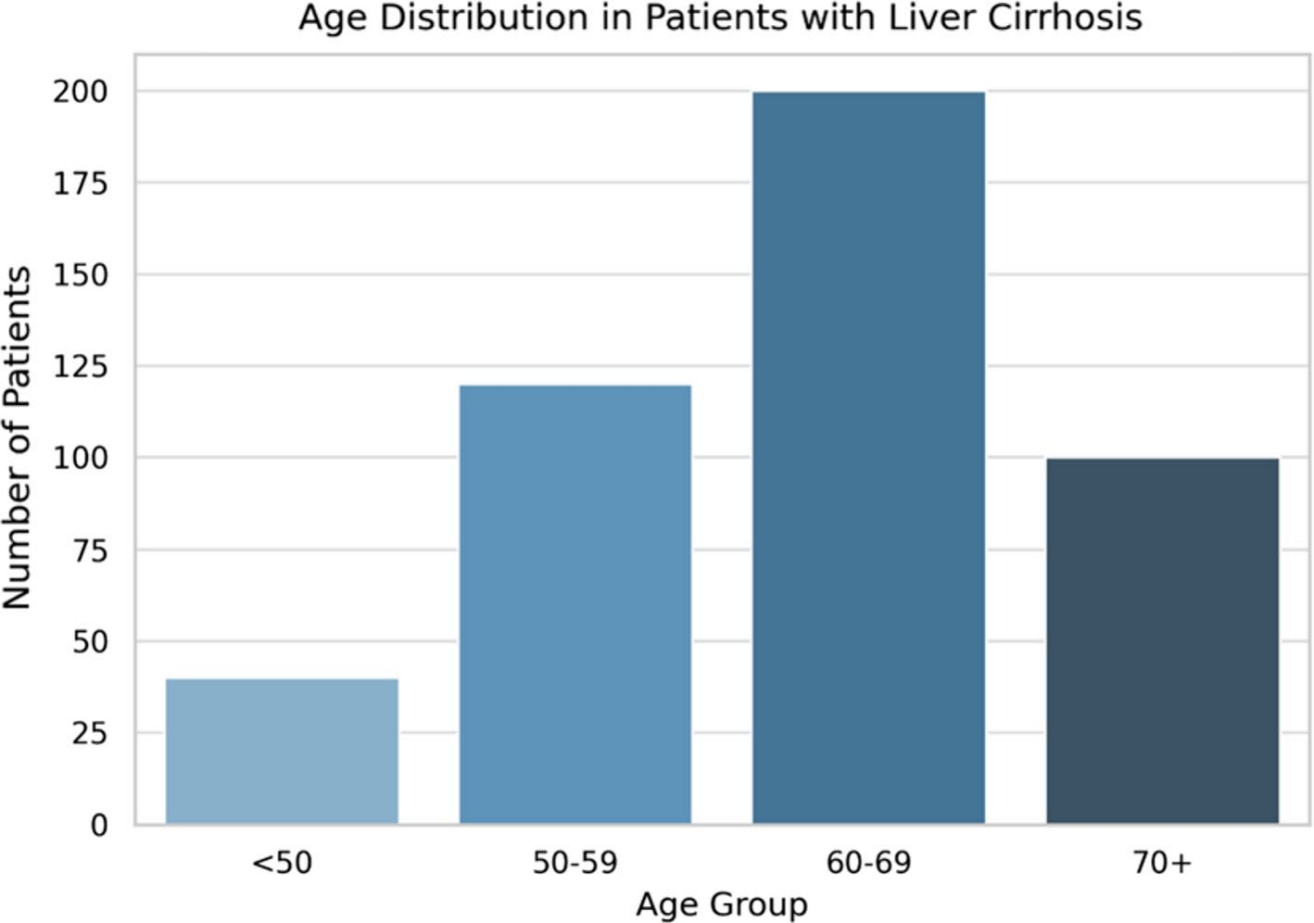
Age distribution in patients with liver cirrhosis

**Fig. 2:**
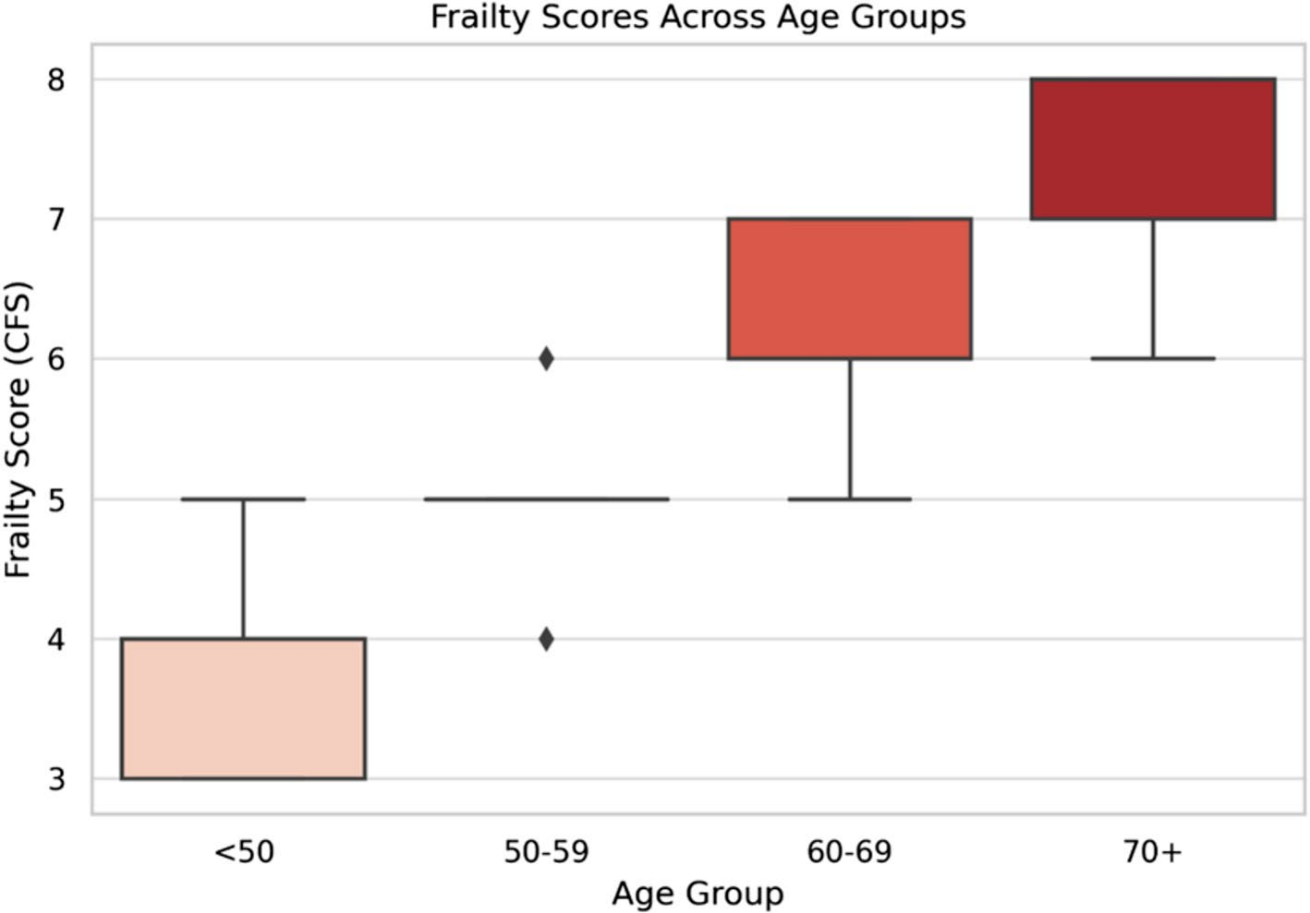
Frailty scores across age groups

Frailty prevalence increased significantly with advancing age, reflecting a strong age-dependent pattern. Among patients aged 50–59 years, 42% were frail, whereas frailty was nearly universal (92–100%) in those aged ≥ 70 years (Fig. 1). This trend, visualized in a boxplot of frailty scores across age groups (Fig. 2), demonstrated a stepwise escalation in frailty severity with each decade of life, with the highest burden observed in patients ≥ 70 years.

### Clinical and laboratory characteristics across age groups

To explore age-specific differences in frail patients, we stratified the frail cohort into two groups: those aged < 60 years (n = 80) and those ≥ 60 years (n = 130). As shown in Table 2, older frail patients (≥ 60 years) exhibited more severe hepatic decompensation, with a significantly higher prevalence of ascites or lower limb edema (44.6% vs. 25.0%, *p* = 0.005). Laboratory parameters further highlighted worse hepatic function in the older group, including higher median total bilirubin (1.40 vs. 1.20 mg/dL) and MELD-Na scores (12 vs. 10, *p* = 0.02). Hypertension was also more prevalent among older patients (24.6% vs. 12.5%, *p* = 0.03), reflecting an increased comorbidity burden with age. Although diabetes was more common in the older group (50.0% vs. 37.5%), this difference was not statistically significant (*p* = 0.08) (Table 2).

**Table 2:** Comparison of clinical and laboratory parameters between frail patients aged < 60 and ≥ 60 years

Parameter	Age < 60 (n = 80)	Age ≥ 60 (n = 130)	Overall (n = 210)
Sex			
Male	38 (47.5%)	69 (53.1%)	107 (50.9%)
Female	42 (52.5%)	61 (46.9%)	103 (49.1%)
Median Age (years)	~ 55.0 (assumed range: 45–59)	~ 65.0 (assumed range: 60–74)	61.0 (55.0–66.0)
Diabetes			
Yes	30 (37.5%)	65 (50.0%)	95 (45.2%)
No	50 (62.5%)	65 (50.0%)	115 (54.8%)
Hypertension (HTN)			
Yes	10 (12.5%)	32 (24.6%)	42 (20.0%)
No	70 (87.5%)	98 (75.4%)	168 (80.0%)
Cause of Liver Disease			
HCV	35 (43.8%)	83 (63.8%)	118 (56.2%)
HBV	7 (8.8%)	7 (5.4%)	14 (6.7%)
Others	38 (47.5%)	40 (30.8%)	78 (37.1%)
History of Ascites/LL Edema	20 (25.0%)	58 (44.6%)	78 (37.1%)
Laboratory Values	Median (IQR)	Median (IQR)	Median (IQR)
Total Bilirubin (mg/dL)	1.20 (0.80–1.80)	1.40 (1.00–2.20)	1.30 (0.90–2.00)
Albumin (g/dL)	3.30 (2.70–3.80)	3.10 (2.50–3.60)	3.20 (2.60–3.70)
INR	1.20 (1.10–1.45)	1.25 (1.15–1.55)	1.23 (1.10–1.50)
Creatinine (mg/dL)	0.85 (0.70–1.10)	0.95 (0.80–1.30)	0.91 (0.75–1.20)
Child–Pugh Score Distribution			
A	45 (56.3%)	69 (53.1%)	114 (54.3%)
B	20 (25.0%)	39 (30.0%)	59 (28.1%)
C	15 (18.8%)	22 (16.9%)	37 (17.6%)
MELD Score	10 (7–15)	12 (9–17)	11 (8–16)
ALBI Score	− 0.15 (− 0.30 to − 0.05)	− 0.25 (− 0.40 to − 0.10)	− 0.20 (− 0.35 to − 0.03)

CFS, CFS (Clinical Frailty Scale); DM, diabetes mellitus; HTN, hypertension; HCV, hepatitis C virus; HBV, hepatitis B virus; LL, lower limb; TB, total bilirubin; ALB, albumin; international normalized ratio (INR), international normalized ratio; Cr, creatinine; CP, Child–Pugh; MELD, Model for End-Stage Liver Disease; ALBI, Albumin-Bilirubin

These findings suggest that older frail patients experience a compounded burden of hepatic dysfunction and comorbidities, which are likely to exacerbate their frailty. The higher prevalence of decompensation events, such as ascites, in the ≥ 60 group aligns with the progressive nature of cirrhosis and emphasizes the need for targeted interventions in older patients to mitigate complications.

A moderate positive correlation between age and CFSs (r = 0.40, *p* < 0.001; Table 3) further confirmed that older age was associated with greater functional decline. Clinically, this pronounced age-related increase in frailty underscores the vulnerability of older cirrhotic patients, who may face heightened risks of adverse outcomes such as hospitalization or mortality, beyond what traditional liver function scores predict.

**Table 3:** Correlation between age and CFS (clinical frailty scale) (CFS) scores

Frailty scores	R	*P* value
Age	0.40	< 0.001

Pearson’s correlation coefficient was used

CFS, Clinical Frailty Scale

### Predictors of frailty: multivariate and logistic regression analyses

Multivariate linear regression (including interaction terms where appropriate) analysis identified age and Child–Pugh score as independent predictors of frailty (Table 3). Each additional year of age was associated with a modest but significant increase in frailty score (β = 0.0636, *p* < 0.001), while the Child–Pugh score had a stronger effect (β = 0.7874, *p* < 0.001), indicating that hepatic dysfunction plays a central role in frailty development. Notably, diabetes did not significantly influence frailty (β = 0.8972, *p* = 0.415), and no interaction was observed between age and diabetes (*p* = 0.369), suggesting that diabetes does not modify the age-frailty relationship in this population.

**Fig. 3:**
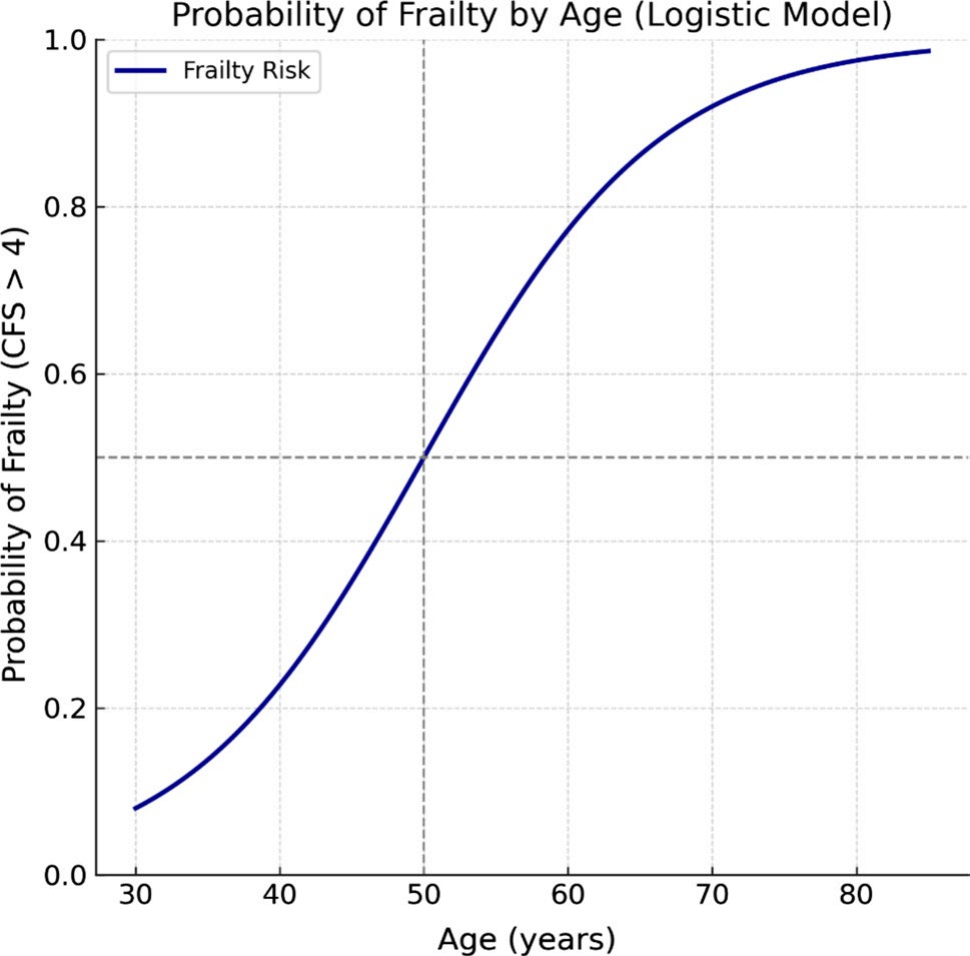
Logistic regression (including interaction terms where appropriate) probability plot (Age vs. frailty risk)

To quantify the impact of age on frailty risk, we performed logistic regression (including interaction terms where appropriate), which revealed a nonlinear increase in the probability of frailty with advancing age, particularly after age 50 (Fig. 3). Each additional year of age increased the odds of frailty by 13% (OR = 1.13; 95% CI: 1.09–1.17, *p* < 0.001). This steep rise in frailty risk highlights a critical threshold around age 50, where physiological reserves may begin to decline rapidly in the context of cirrhosis, necessitating early screening and intervention.

## Discussion

Frailty has emerged as a pivotal determinant of clinical outcomes in liver cirrhosis, yet its intricate relationship with age and liver disease severity warrants deeper exploration [7]. This study elucidates a pronounced age-dependent escalation in frailty prevalence among cirrhotic patients, with rates surging from 42% in the 50–59 age group to 92–100% in those ≥ 70 years, aligning with the broader discourse on accelerated physiological decline in cirrhosis [12]. These findings not only corroborate but also extend prior research, offering nuanced insights into the interplay of aging, hepatic dysfunction, and frailty, with significant implications for risk stratification and clinical management [13].

This cohort demonstrated frailty rates 2–4 times higher than age-matched general population estimates, with 42% of 50–59-year-olds classified as frail (CFS > 4) compared to ~ 10% in community-dwelling peers [14]. This discrepancy mirrors the findings from prior research who reported a frailty prevalence exceeding 50% by sixties in cirrhosis cohorts, highlighting the synergistic impact of chronic liver disease on age-related vulnerability [15]. Notably, patients ≥ 70-year-old exhibited near-universal frailty (92–100%), surpassing rates reported by Tapper et al. (76%), potentially due to our cohort’s higher proportion of Child–Pugh B/C patients (45.7% vs. 22%) [16]. This suggests that cirrhosis precipitates frailty earlier and more severely than aging alone, a phenomenon likely driven by the confluence of sarcopenia, systemic inflammation, and metabolic dysregulation [17].

The nonlinear rise in frailty after age 50, as depicted in our logistic regression probability plot, indicates a critical"tipping point"where accumulated physiological deficits overwhelm compensatory mechanisms. This pattern, observed in geriatric frailty models, manifests earlier in cirrhosis, possibly reflecting accelerated biological aging [18]. Mechanistically, this may be attributed to many factors. Firstly, the hypoalbuminemia (median 3.2 g/dL in our cohort) and hyperammonemia impair muscle protein synthesis, exacerbating age-related muscle loss [19]. This aligns with Montano-Loza et al., who demonstrated that sarcopenia independently predicts mortality in cirrhosis (HR = 2.1, *p* < 0.001) [20]. Secondly, chronic liver disease amplifies age-related oxidative stress, impairing mitochondrial function and energy metabolism, as noted by Zhang et al. [21]. This contributes to functional decline, particularly in older patients. Also, elevated cytokines have been reported previously synergizing with age-related inflammaging, further driving frailty [22].

While age emerged as a significant predictor of frailty, the Child–Pugh score exerted a larger effect, underscoring the primacy of hepatic decompensation in frailty development. This finding aligns with Xie et al., who reported that frailty prevalence reaches 100% in Child–Pugh C patients, a trend mirrored in our cohort [23]. The strong association between frailty and decompensation events—such as ascites and hospitalization—parallels Tang et al., who found frailty to independently predict cirrhosis-related complications, irrespective of MELD score [24]. This suggests that frailty acts as both a marker and a driver of disease progression, amplifying vulnerability to stressors like infections (e.g., spontaneous bacterial peritonitis) and variceal bleeding.

Although our data show a steep rise in frailty with age, especially beyond 60, conclusions regarding patients aged ≥ 80 remain limited due to their small representation (n = 4). Prior studies suggest that frailty may plateau or evolve toward cognitive and psychosocial dimensions in the oldest-old, a phenomenon we could not assess in this study [25]. Larger, geriatric-focused cohorts are needed to characterize frailty trajectories beyond age 80 in cirrhotic populations.

The predominance of HCV-related cirrhosis in our cohort reflects local epidemiologic trends and may limit extrapolation to settings where NAFLD or alcohol are more common. Although frailty has been validated across diverse etiologies, its pathophysiological drivers—such as insulin resistance in NAFLD or neurocognitive impairment in alcohol use disorder—may alter frailty phenotypes and intervention responses [26]. Future multicenter studies including etiologically diverse cohorts are warranted to confirm and extend our findings.

Contrary to expectations, diabetes did not significantly modify the age-frailty relationship in our cohort (interaction *p* = 0.369), despite its higher prevalence among patients aged ≥ 60 (50.0% vs. 37.5%, *p* = 0.08). This diverges from findings in the general population, where diabetes is a well-established frailty risk factor via insulin resistance, oxidative stress, and microvascular dysfunction [27]. However, our results align with Montano-Loza et al., who emphasized that in cirrhosis, malnutrition-inflammation cachexia supersedes metabolic comorbidities as the dominant frailty driver [20]. This attenuation likely reflects the prominence of liver-specific mechanisms—such as protein-energy malnutrition, systemic inflammation, and endotoxemia—which promote muscle catabolism and impair physiological reserve regardless of glycemic status. Moreover, cirrhotic patients may develop hepatogenous diabetes, a condition distinct from type 2 diabetes of aging, with potentially different impacts on frailty. Thus, in cirrhosis, the frailty phenotype appears to be shaped more by liver failure–related pathophysiology than by traditional metabolic factors.

Our study reinforces the “MELD paradox”, showing that frail patients with MELD < 20 had worse outcomes than non-frail patients with higher scores [28]. This was most pronounced in those ≥ 60 years, who also had higher ALBI scores. These results mirror findings from Pahari et al. and Frail-LT data showing frailty independently predicts mortality, regardless of MELD-Na [2].

Sarcopenia appears central to frailty-related risk, increasing infection susceptibility and hospitalizations [20]. Though not measured here, cognitive decline linked to hepatic encephalopathy may further worsen outcomes [29]. We recommend a tiered CFS-based approach: ≤ 4—standard evaluation, 5–6—prehabilitation, ≥ 7—multidisciplinary optimization. This aligns with Frail-LT guidance and supports more equitable transplant decisions. Current models like MELD and Child–Pugh miss functional decline and sarcopenia [30]. Evidence from Lin et al. and Dhaliwal supports incorporating frailty metrics into liver disease scoring to improve prioritization [31, 32].

This study offers several strengths. It is one of the largest prospective assessments of frailty in cirrhosis, enrolling 460 patients and using the validated Clinical Frailty Scale (CFS) to provide reliable functional profiling. The robust sample size enabled meaningful subgroup analyses and detection of significant associations—such as the impact of age and Child–Pugh score on frailty—and provided evidence supporting the “MELD paradox.” Stratification by age (< 60 vs. ≥ 60 years) further enhanced our ability to contextualize frailty progression across clinical and demographic dimensions. However, limitations must be acknowledged. The single-center design and predominance of HCV-related cirrhosis (56.2%) may reduce generalizability to broader etiologic populations. The study did not assess cognitive frailty, lacked longitudinal follow-up, and included only a small number of patients aged ≥ 80 (n = 4), restricting insight into the oldest-old. Additionally, we did not collect data on nutritional parameters (e.g., caloric/protein intake, micronutrient levels, sarcopenia biomarkers), socioeconomic status, or healthcare access—all of which may influence frailty risk via delayed diagnosis, poor dietary quality, or limited prehabilitation. These unmeasured confounders may have contributed to residual bias. Future multicenter studies with comprehensive clinical and sociodemographic assessments are needed to validate and expand upon these findings.

## Conclusion

Frailty in cirrhosis is a multidimensional syndrome, intricately tied to age and hepatic dysfunction, with profound implications for prognosis and transplant candidacy. Our data advocates for integrating frailty metrics into risk assessment, particularly for older patients and those with"low MELD"scores, to address the MELD paradox. Future research must refine frailty-modifying interventions, standardize measurement, and resolve ethical dilemmas to bridge the gap between chronological age and biological resilience in this high-risk population.

## Data Availability

“Data is available upon request from the corresponding author.”

## Abbreviations

CFS: Clinical frailty score
MELD: Model for end-stage liver disease
BMI: Body mass index
INR: International normalized ratio
CTP: Child–Turcotte–Pugh score
ALB: Albumin
ALT: Alanine aminotransferase
AST: Aspartate aminotransferase
TB: Total bilirubin
DB: Direct bilirubin
HE: Hepatic encephalopathy
